# Ampelopsin Inhibits Cell Proliferation and Induces Apoptosis in HL60 and K562 Leukemia Cells by Downregulating AKT and NF-κB Signaling Pathways

**DOI:** 10.3390/ijms22084265

**Published:** 2021-04-20

**Authors:** Jang Mi Han, Hong Lae Kim, Hye Jin Jung

**Affiliations:** 1 Department of Life Science and Biochemical Engineering, Sun Moon University, Asan 31460, Korea; gkswkdal200@naver.com; 2 Department of Pharmaceutical Engineering and Biotechnology, Sun Moon University, Asan 31460, Korea; llee5405@gmail.com; 3 Genome-Based BioIT Convergence Institute, Asan 31460, Korea

**Keywords:** leukemia, ampelopsin, proliferation, apoptosis, cancer stemness

## Abstract

Leukemia is a type of blood cancer caused by the rapid proliferation of abnormal white blood cells. Currently, several treatment options, including chemotherapy, radiation therapy, and bone marrow transplantation, are used to treat leukemia, but the morbidity and mortality rates of patients with leukemia are still high. Therefore, there is still a need to develop more selective and less toxic drugs for the effective treatment of leukemia. Ampelopsin, also known as dihydromyricetin, is a plant-derived flavonoid that possesses multiple pharmacological functions, including antibacterial, anti-inflammatory, antioxidative, antiangiogenic, and anticancer activities. However, the anticancer effect and mechanism of action of ampelopsin in leukemia remain unclear. In this study, we evaluated the antileukemic effect of ampelopsin against acute promyelocytic HL60 and chronic myelogenous K562 leukemia cells. Ampelopsin significantly inhibited the proliferation of both leukemia cell lines at concentrations that did not affect normal cell viability. Ampelopsin induced cell cycle arrest at the sub-G1 phase in HL60 cells but the S phase in K562 cells. In addition, ampelopsin regulated the expression of cyclins, cyclin-dependent kinases (CDKs), and CDK inhibitors differently in each leukemia cell. Ampelopsin also induced apoptosis in both leukemia cell lines through nuclear condensation, loss of mitochondrial membrane potential, increase in reactive oxygen species (ROS) generation, activation of caspase-9, caspase-3, and poly ADP-ribose polymerase (PARP), and regulation of Bcl-2 family members. Furthermore, the antileukemic effect of ampelopsin was associated with the downregulation of AKT and NF-κB signaling pathways. Moreover, ampelopsin suppressed the expression levels of leukemia stemness markers, such as Oct4, Sox2, CD44, and CD133. Taken together, our findings suggest that ampelopsin may be an attractive chemotherapeutic agent against leukemia.

## 1. Introduction

Leukemia is a heterogeneous group of malignant disorders in which the number of immature or incompletely differentiated leukocytes increases in the blood or bone marrow. Leukemia represents a major cause of cancer-related deaths worldwide [1,2]. Acute myeloid leukemia (AML) and chronic myeloid leukemia (CML) are the main subgroups of leukemia [3,4]. AML is an aggressive malignancy characterized by abnormal proliferation of myeloid progenitor cells and is strongly associated with mutations in the *FMS-like tyrosine kinase 3 (FLT3)* gene or the abnormal fusion gene *promyelocytic leukemia/retinoic acid receptor alpha (PML/RARA)* [3,5]. CML is a clonal stem cell disease characterized by the proliferation of granulocytes at all stages of differentiation and is driven by the Philadelphia chromosome, which generates an active chimeric BCR-ABL tyrosine kinase [4,6]. Therefore, aberrantly activated FLT3 and BCR-ABL tyrosine kinases and PML/RARα fusion protein have emerged as promising molecular targets for the treatment of AML and CML. Several tyrosine kinase inhibitors (TKIs), such as FLT3 inhibitors (sorafenib and midostaurin) and BCR-ABL inhibitors (imatinib and nilotinib), and PML/RARα inhibitors (all-*trans* retinoic acid and arsenic trioxide) have been developed and clinically applied to patients with AML and CML as single agents or in combination with chemotherapy [7,8,9,10]. However, the most definitive and effective treatment for leukemia is bone marrow transplantation [11]. Unfortunately, the insufficient response to molecular targeted drugs, the low success ratio of bone marrow transplantation, acquired drug resistance, and serious adverse effects of therapies remain major therapeutic challenges [7,8,9,10,11]. Therefore, there is an unmet need to develop more selective and less toxic drugs for the effective treatment of leukemia.

Apoptosis, a type of programmed cell death, is required for the homeostasis of normal cells and is associated with diverse cellular properties, such as cell shrinkage, decreased mitochondrial membrane potential, chromatin condensation, and DNA fragmentation [12,13]. Apoptosis is mediated by caspases, which are activated by intrinsic and extrinsic pathways, and regulated by various intracellular molecular mechanisms [13]. Dysregulation of apoptosis plays an important role in the development of various human diseases, including cancer [13]. The evasion of apoptosis is regarded as one of the hallmarks of cancer [13,14]. Tumor cells can suppress apoptosis by activating major anti-apoptotic pathways, such as phosphatidylinositol 3-kinase/protein kinase B (PI3K/AKT) and nuclear factor-kappa B (NF-κB) [15,16]. The PI3K/AKT and NF-κB pathways are aberrantly upregulated in various cancers, including leukemia [15,16]. PI3K/AKT signaling plays a key role in regulating the metabolism, growth, proliferation, and survival of tumor cells [15]. NF-κB is a transcription factor that regulates the expression of diverse genes associated with tumor cell growth and survival [16]. Therefore, effective inhibition of these pathways could be a promising therapeutic strategy to suppress leukemia cell growth by promoting apoptosis.

Plant-derived natural compounds have gained tremendous attention owing to their broad spectrum of health benefits, safety, and low side effects [17]. Flavonoids are a ubiquitous group of polyphenolic secondary metabolites found in plants [18]. Epidemiological, clinical, and animal studies have revealed that flavonoids exert protective effects against various medical disorders, including cardiovascular disease and cancer [19,20,21]. In particular, flavonoids have been shown to suppress proliferation, angiogenesis, and metastasis and increase apoptosis of various cancer cells by interfering with multiple signal transduction pathways [20,21]. Ampelopsin, also known as dihydromyricetin, is a flavonoid found in several medicinal plants, including *Ampelopsis* species and *Hovenia dulcis* [22]. Ampelopsin has been reported to possess multiple pharmacological functions, such as antioxidative, anti-inflammatory, antimicrobial, antiangiogenic, and anticancer activities [23,24,25,26,27]. Previous studies have demonstrated that ampelopsin inhibits proliferation, promotes apoptosis, and suppresses the invasion and migration of several cancer cells, including hepatocellular carcinoma, non-small cell lung cancer, osteosarcoma, ovarian cancer, gastric cancer, breast cancer, melanoma, and glioma, by regulating the mitogen-activated protein kinase (MAPK), AMP-activated protein kinase (AMPK), and PI3K/AKT pathways [27,28,29,30,31,32,33,34]. However, its chemotherapeutic effect on leukemia and underlying mechanism of action remains unclear.

In the present study, the effect of ampelopsin on cell proliferation and apoptosis in HL60 AML and K562 CML cells was assessed. Our results demonstrated that ampelopsin effectively suppressed proliferation and induced apoptosis in both the leukemia cell lines by downregulating the AKT and NF-κB pathways. Therefore, these findings suggest that ampelopsin might be an attractive chemotherapeutic agent against leukemia, such as AML and CML.

## 2. Results

### 2.1. Ampelopsin Inhibits Proliferation of Leukemia Cells

**Figure 1:**
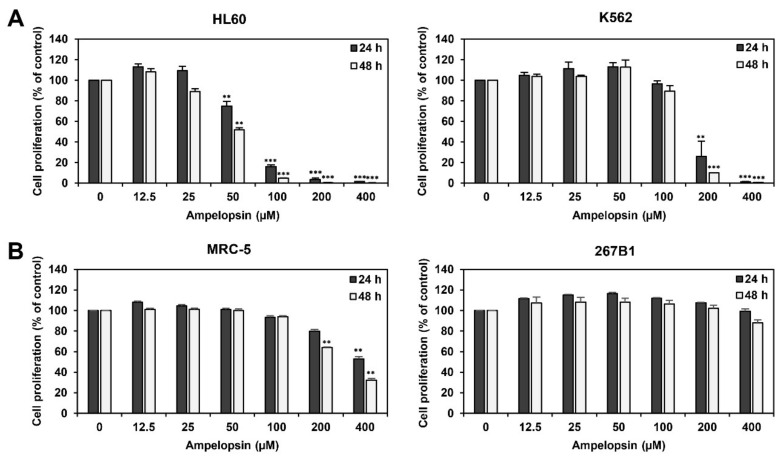
Effect of ampelopsin on the proliferation of leukemia cells. (**A**) Effect of ampelopsin on the proliferation of HL60 and K562 leukemia cells. Cells were treated with increasing concentrations of ampelopsin (0−400 µM) for 24 and 48 h. (**B**) Effect of ampelopsin on the proliferation of MRC-5 and 267B1 normal cells. Cells were treated with ampelopsin (0−400 µM) for 24 and 48 h. (**A**,**B**) Cell proliferation was measured using a CellTiter-Glo^®^ luminescent assay system. ** *p* < 0.005, *** *p* < 0.001 vs. the control.

To examine whether ampelopsin affects the proliferation of leukemia cells, human HL60 AML and K562 CML cell lines were treated with ampelopsin (0–400 µM) for 24 or 48 h. Cell proliferation was then evaluated using the ATP-monitoring luminescence assay. As shown in Figure 1A, ampelopsin treatment inhibited the proliferation of HL60 and K562 cells in a dose-dependent manner. The IC_50_ values of ampelopsin for HL60 and K562 cells were 60.77 and 156.2 µM at 24 h and 45.1 and 135.2 µM at 48 h, respectively, indicating that ampelopsin more potently inhibited the proliferation of HL60 cells than that of K562 cells.

We next evaluated the effect of ampelopsin on the proliferation of two human normal cell lines, MRC-5 fetal lung fibroblast and 267B1 prostate epithelial cells. Ampelopsin inhibited the proliferation of MRC-5 and 267B1 cells with IC_50_ values of 417.1 and >400 µM at 24 h and 276.2 and >400 µM at 48 h after treatment, respectively (Figure 1B). These data imply that ampelopsin suppresses the proliferation of leukemia cells much more sensitively than normal cells.

### 2.2. Ampelopsin Induces Cell Cycle Arrest of Leukemia Cells

**Figure 2:**
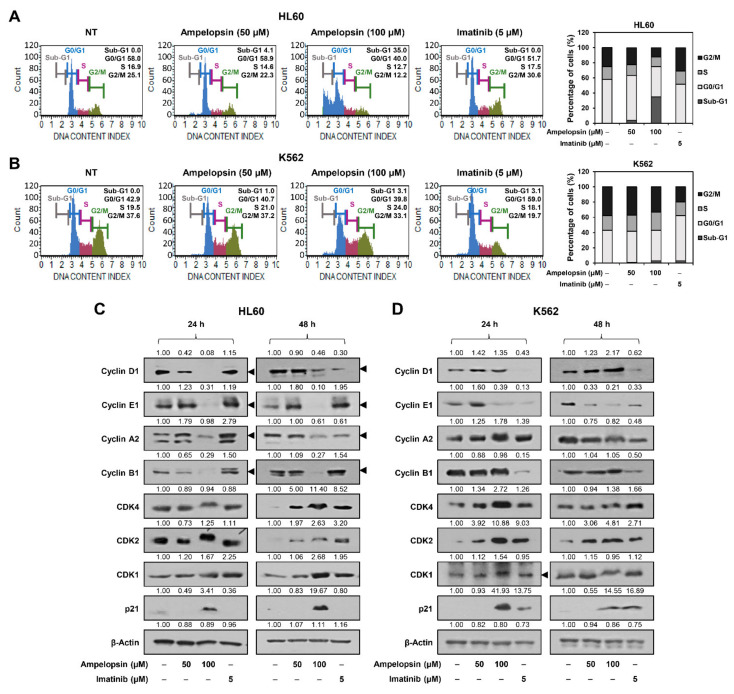
Effect of ampelopsin on the cell cycle of leukemia cells. (**A**,**B**) The cell cycle distribution of HL60 and K562 cells was evaluated using a Muse cell analyzer after treatment with ampelopsin (50 and 100 μM) or imatinib (5 µM) for 24 h. (**C**,**D**) Effect of ampelopsin on the expression of cell cycle regulators in HL60 and K562 cells. Cells were treated with ampelopsin (50 and 100 μM) or imatinib (5 µM) for 24 and 48 h. Protein levels were detected by western blot analysis using specific antibodies and further quantified by densitometry. β-actin levels were used as an internal control. Arrowheads indicate bands that correspond to specific proteins.

We further investigated the anticancer effect of ampelopsin on leukemia cells at concentrations that did not affect normal cell growth (50 and 100 µM). To evaluate whether ampelopsin inhibits the proliferation of leukemia cells by regulating the cell cycle, we examined the effect of ampelopsin on cell cycle distribution using the Muse cell analyzer. Imatinib, a tyrosine kinase inhibitor of BCR-ABL used as the standard first-line therapy for CML, was used as a control drug [7]. Compared with the untreated control cells, treatment with ampelopsin for 24 h significantly increased the sub-G1 phase cell population in HL60 AML cells, whereas imatinib mildly increased the cell population in the G2/M phase (Figure 2A). However, ampelopsin mildly increased the S phase cell population in K562 CML cells compared to that in the untreated control cells, whereas imatinib markedly increased the G0/G1 phase cell population (Figure 2B). Therefore, ampelopsin arrested cell cycle progression at the sub-G1 phase in HL60 cells and S phase in K562 cells. In addition, ampelopsin induced cell cycle arrest in HL60 cells more strongly than in K562 cells. As previously reported [35], imatinib prominently caused cell cycle arrest in K562 CML cells at the G0/G1 phase.

Cell cycle checkpoint proteins, such as cyclins and cyclin-dependent kinases (CDKs), play an important role in regulating cell cycle progression [36]. We next determined whether ampelopsin affects the expression of the cell cycle regulators in HL60 and K562 leukemia cells. As shown in Figure 2C, in HL60 cells, ampelopsin significantly reduced the levels of cyclin proteins, including cyclin D1, cyclin E1, cyclin A2, and cyclin B1, at both 24 and 48 h of treatment, whereas imatinib decreased the levels of cyclin D1 and cyclin A2 after 48 h of treatment. However, ampelopsin and imatinib did not reduce the levels of CDK proteins, including CDK4, CDK2, and CDK1, after 24 h of treatment, whereas both compounds increased the levels of CDK proteins after 48 h of treatment. Furthermore, ampelopsin markedly induced the expression of a CDK inhibitor, p21, but imatinib did not. These data indicate that ampelopsin-mediated sub-G1 phase arrest in HL60 cells may be associated with the downregulation of cyclins and upregulation of p21.

In K562 cells, ampelopsin reduced the level of cyclin E1 at both 24 and 48 h of treatment, but not that of cyclin D1, cyclin A2, and cyclin B1 (Figure 2D). Imatinib significantly decreased the levels of cyclin D1, cyclin E1, and cyclin B1 at both 24 and 48 h of treatment, whereas it did not affect the level of cyclin A2. However, ampelopsin and imatinib did not reduce the CDK protein levels. The expression of p21 was increased by both the compounds. These data indicate that the ampelopsin-induced S phase arrest in K562 cells may be related to the regulation of cyclin E1 and p21 expression. Taken together, the cell cycle arrest through different phases by ampelopsin in HL60 AML and K562 CML cells may be attributed to the different regulation of the expression of cyclins, CDKs, and CDK inhibitors depending on the subtypes of leukemia cells.

### 2.3. Ampelopsin Induces Apoptosis in Leukemia Cells

**Figure 3:**
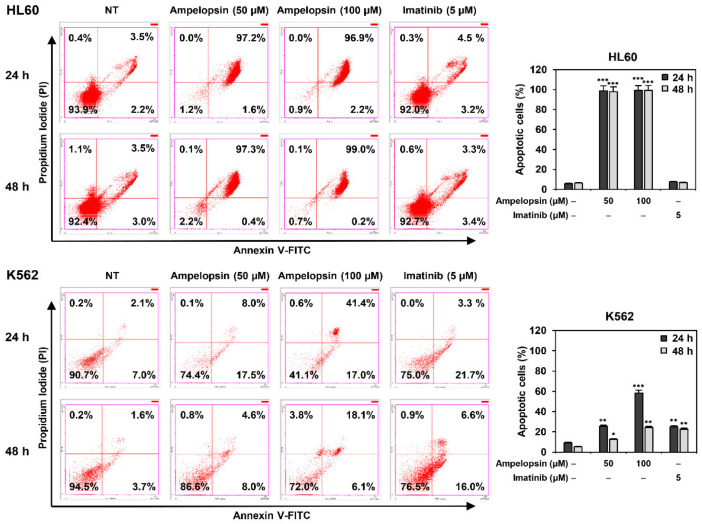
Effect of ampelopsin on the apoptotic cell death of leukemia cells. HL60 and K562 cells were treated with ampelopsin (50 and 100 µM) or imatinib (5 µM) for 24 and 48 h. Apoptotic cells were determined by flow cytometry following Annexin V-FITC and propidium iodide (PI) dual labeling. * *p* < 0.05, ** *p* < 0.005, *** *p* < 0.001 vs. the control.

To further elucidate the antiproliferative effect of ampelopsin in leukemia cells, cellular apoptosis was quantitatively measured using flow cytometry following Annexin V-FITC and PI dual labeling. As shown in Figure 3, treatment with ampelopsin for 24 and 48 h markedly increased the total number of early and late apoptotic cells in HL60 cells compared to that of untreated control cells, while imatinib did not. Ampelopsin also increased the proportion of early and late apoptotic cells at both 24 and 48 h of treatment in K562 cells, but its apoptosis-inducing effect was stronger in HL60 AML cells than in K562 CML cells. In contrast, imatinib significantly induced apoptosis in K562 CML cells but not in HL60 AML cells.

**Figure 4:**
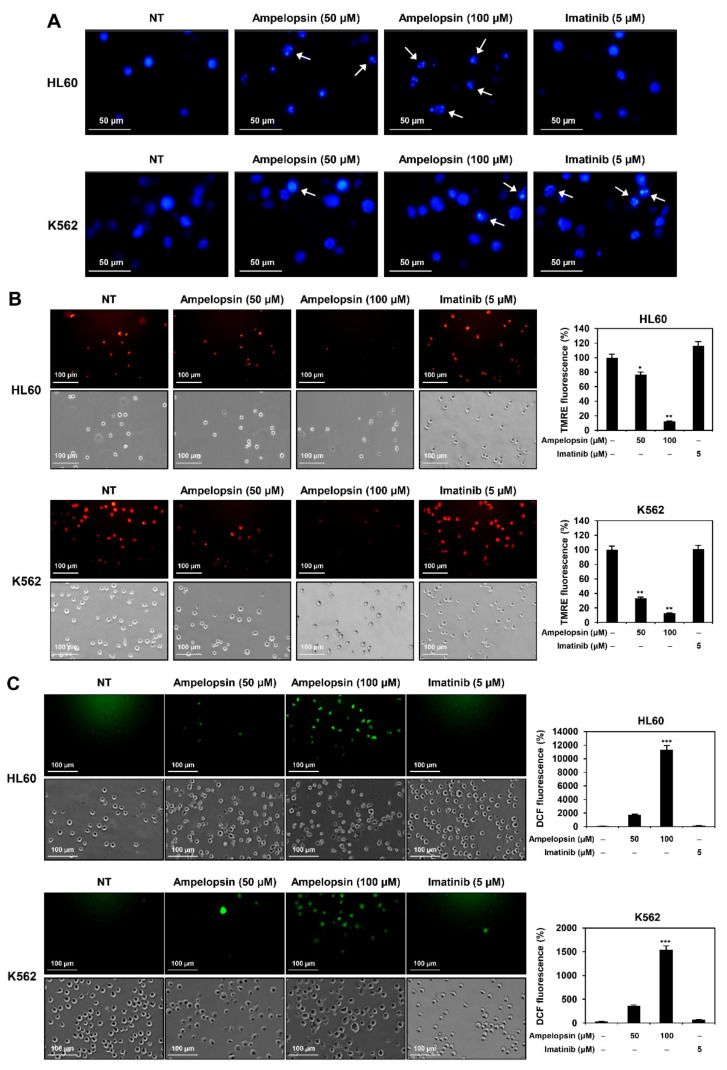

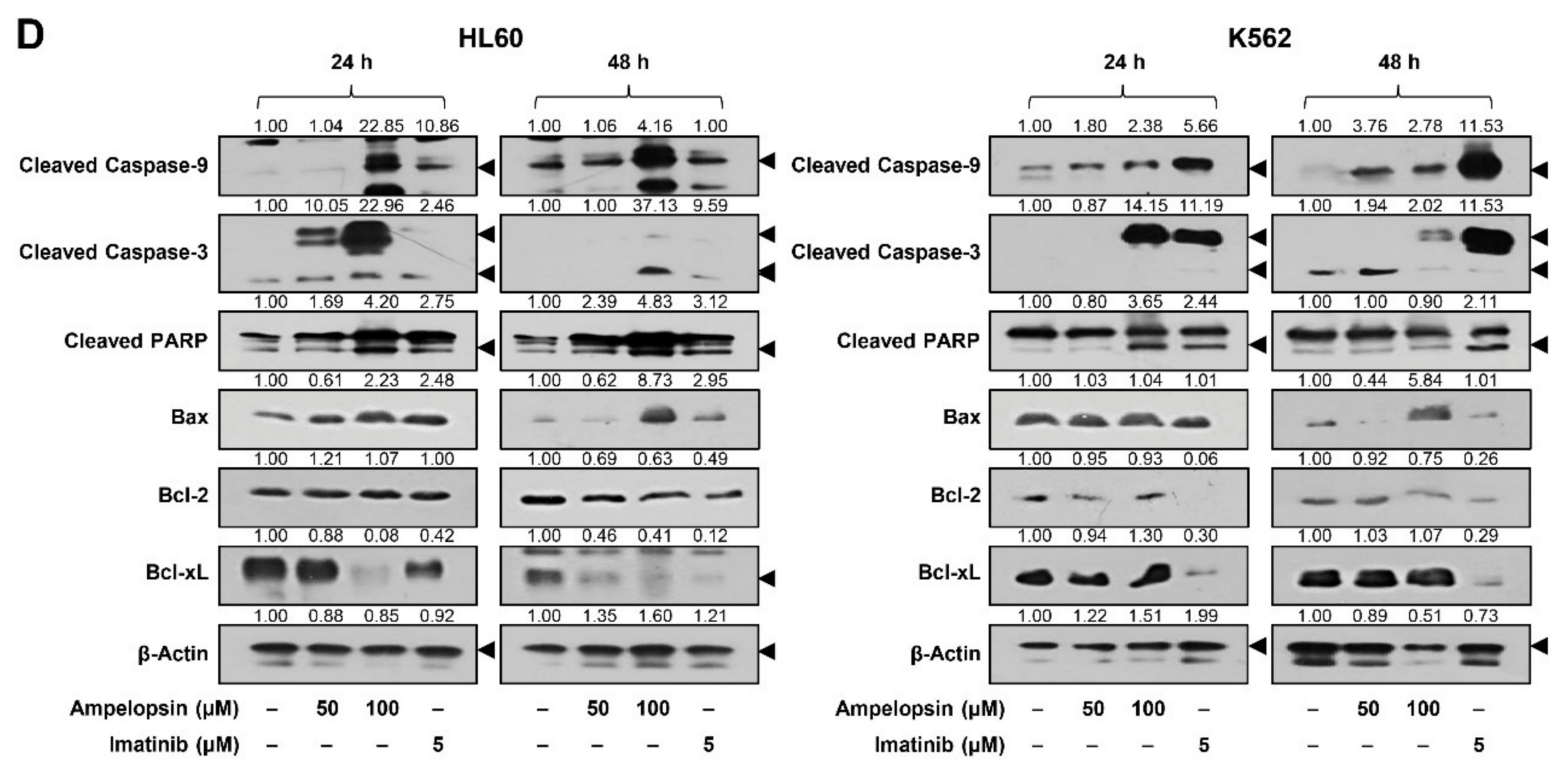
Effect of ampelopsin on the mitochondria-mediated apoptosis of leukemia cells. (**A**) Effect of ampelopsin on the nuclear morphology. HL60 and K562 cells were treated with ampelopsin (50 and 100 µM) or imatinib (5 µM) for 24 h. Changes in nuclear morphology were monitored by DAPI staining under a fluorescence microscope. The condensed and fragmented nuclei were indicated by white arrows. (**B**) Effect of ampelopsin on the mitochondrial membrane potential. Leukemia cells were treated with ampelopsin or imatinib for 6 h and stained with TMRE. Fluorescence images were obtained with a fluorescence microscope and further quantified by densitometry. (**C**) Effect of ampelopsin on the intracellular ROS generation. Leukemia cells were treated with ampelopsin or imatinib for 6 h. ROS levels were detected with DCFH-DA using a fluorescence microscope and further quantified by densitometry. (**D**) Effect of ampelopsin on the mitochondria-mediated apoptotic pathway. Leukemia cells were treated with ampelopsin or imatinib for 24 and 48 h. Protein levels were detected by western blot analysis using specific antibodies and further quantified by densitometry. β-actin levels were used as an internal control. Arrowheads indicate bands that correspond to specific proteins. * *p* < 0.05, ** *p* < 0.005, *** *p* < 0.001 vs. the control.

To further characterize the apoptosis induced by ampelopsin in leukemia cells, we investigated whether it causes nuclear apoptotic changes in HL60 and K562 cells. DAPI staining showed that treatment with ampelopsin for 24 h induced nuclear condensation and fragmentation of both cells (Figure 4A). However, nuclear alterations by ampelopsin were observed more clearly in HL60 cells than in K562 cells, whereas those by imatinib were observed in K562 cells, but not in HL60 cells.

Mitochondria play a key role in activating apoptosis [13]. Because the loss of mitochondrial membrane potential (MMP) is an early event in the apoptotic process [13], we next determined whether ampelopsin leads to the depolarization of MMP in leukemia cells by tetramethylrhodamine ethyl ester (TMRE) staining. TMRE is a red-orange fluorescent dye that accumulates in the mitochondria in proportion to MMP. As shown in Figure 4B, the control cells exhibited a high red fluorescence, whereas treatment with ampelopsin for 6 h decreased red fluorescence in HL60 and K562 cells in a dose-dependent manner, indicating that ampelopsin significantly led to the loss of MMP in both leukemia cell lines. However, imatinib treatment for 6 h did not cause any changes in MMP in either cell line.

Generation of reactive oxygen species (ROS) is closely related to the induction of apoptosis mediated by mitochondria, death receptors, and endoplasmic reticulum (ER) [13]. We thus examined whether ampelopsin affects ROS generation in HL60 and K562 cells after 6 h of treatment using the fluorescent DCFH-DA product. Ampelopsin remarkably elevated the generation of ROS in HL60 and K562 cells compared to that in control cells, but imatinib did not (Figure 4C). These results imply that the induction of ROS is involved in the regulation of apoptosis caused by ampelopsin in both leukemia cell lines.

We next assessed whether ampelopsin regulates the mitochondria-mediated apoptotic pathway in leukemia cells. HL60 and K562 cells were treated with ampelopsin for 24 and 48 h, and the protein levels of caspases and Bcl-2 family members were measured. As shown in Figure 4D, treatment with ampelopsin increased the expression levels of cleaved caspase-9, cleaved caspase-3, and cleaved PARP in HL60 and K562 cells. Furthermore, the level of the pro-apoptotic protein Bax was upregulated by ampelopsin treatment, and the expression of the anti-apoptotic protein Bcl-xL, but not Bcl-2, was downregulated in HL60 cells. However, in K562 cells, ampelopsin induced the expression of Bax, whereas it did not affect that of Bcl-2 and Bcl-xL. Meanwhile, imatinib significantly elevated the expression of cleaved caspase-9, cleaved caspase-3, and cleaved PARP, as well as more effectively inhibited the levels of Bcl-2 and Bcl-xL in K562 cells than in HL60 cells. These results suggest that ampelopsin induces apoptosis via the regulation of the mitochondrial apoptotic pathway in HL60 AML and K562 CML cells.

### 2.4. Ampelopsin Downregulates AKT and NF-κB Signaling in Leukemia Cells

**Figure 5:**
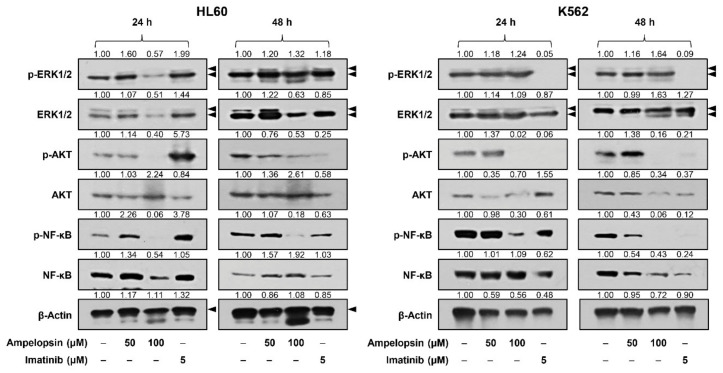
Effect of ampelopsin on the ERK1/2, AKT, and NF-κB signaling in leukemia cells. HL60 and K562 cells were treated with ampelopsin (50 and 100 μM) or imatinib (5 µM) for 24 and 48 h. Protein levels were detected by western blot analysis using specific antibodies and further quantified by densitometry. β-actin levels were used as an internal control. Arrowheads indicate bands that correspond to specific proteins.

The aberrant activation of MAPK/extracellular signal kinase (ERK), PI3K/AKT, and NF-κB signaling pathways have been implicated in the pathogenesis of leukemia [15,16,37]. Thus, we evaluated whether ampelopsin affects these oncogenic pathways in HL60 and K562 cells. As shown in Figure 5, treatment with ampelopsin for 24 and 48 h more effectively suppressed the expression levels of phosphorylated forms compared to unphosphorylated AKT and NF-κB proteins in both cell lines. However, ampelopsin did not inhibit the phosphorylation of ERK1/2. Imatinib treatment significantly inhibited the phosphorylation of ERK1/2, AKT, and NF-κB in K562 cells but not in HL60 cells. These results suggest that the inhibitory effect of ampelopsin on leukemia cell growth may be associated with the downregulation of AKT and NF-κB signaling pathways.

### 2.5. Ampelopsin Inhibits the Expression of Stemness Markers in Leukemia Cells

**Figure 6:**
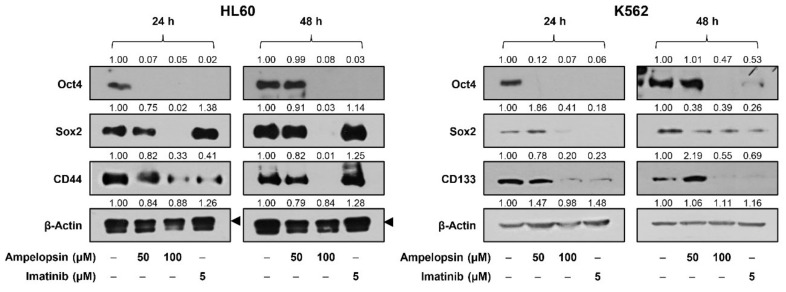
Effect of ampelopsin on the expression of stemness markers in leukemia cells. HL60 and K562 cells were treated with ampelopsin (50 and 100 μM) or imatinib (5 µM) for 24 and 48 h. ProTable.

Accumulating evidence has revealed that leukemia stem cells (LSCs) facilitate chemoresistance and relapse of leukemia [38]. Accordingly, we further confirmed the effect of ampelopsin on the stemness of leukemia cells. As shown in Figure 6, ampelopsin treatment effectively reduced the expression levels of the key stemness transcription factors, Oct4 and Sox2, as well as the cell surface markers for LSCs, CD44 and CD133, in HL60 and K562 cells. However, imatinib more sensitively inhibited the expression of the stemness markers in K562 cells than HL60 cells. These data imply that ampelopsin promotes apoptosis and obstructs the stemness of leukemia cells.

## 3. Discussion

Drug resistance and metastasis are the leading causes of treatment failure in patients with leukemia, and it is difficult to eradicate all aggressive leukemia cells with traditional treatments [1,39]. Therefore, the development of new chemotherapeutic agents with high efficacy and low toxicity is urgently needed to improve the treatment outcomes of leukemia. Increasing reports have demonstrated the chemopreventive and chemotherapeutic potential of diverse plant-derived compounds against leukemia [39]. Flavonoids are plant secondary metabolites that have been recognized as a great source of novel chemotherapeutic drug candidates because of their low side effects and multiple cellular mechanisms of action [17]. Various types of flavonoids, such as apigenin, quercetin, naringenin, genistein, and hesperidin, have exhibited antiproliferative and cytotoxic effects in human leukemia cell lines through the regulation of diverse cellular signaling pathways and molecular mechanisms [40]. In addition, several flavonoids possess superior therapeutic potential in chemoresistant leukemia cells compared to their parent cells [40,41]. Therefore, flavonoids may be developed as promising antileukemic drugs as single agents or in combination with chemotherapy.

In the present study, we investigated the anticancer effects and underlying molecular mechanisms of ampelopsin, a flavanonol, on leukemia. Several previous studies have shown that co-treatment with ampelopsin and ondansetron enhanced the antiproliferative effect of adriamycin in adriamycin-resistant K562 CML cells by downregulating soluble resistance-related calcium-binding protein (SORCIN) [42]. Ampelopsin also promoted the anticancer activity of adriamycin in U937 AML cells in a p53-dependent manner [43]. In addition, ampelopsin and all-*trans* retinoic acid combination treatment synergistically induced the differentiation of NB4 AML cells through signal transducer and activator of transcription 1 (STAT1) activation [44]. However, the antileukemic activity of ampelopsin alone remains unclear.

Our results revealed that ampelopsin induces cell proliferation inhibition and apoptosis in HL60 AML and K562 CML cells by downregulating AKT and NF-κB signaling pathways. Ampelopsin significantly inhibited the proliferation of both leukemia cell lines without affecting normal cell viability. It induced cell cycle arrest at the sub-G1 phase in HL60 cells and the S phase in K562 cells. The different responses to ampelopsin treatment of both leukemia cells were associated with a difference in cell cycle checkpoint regulators affected by the compound. In HL60 AML cells, the ampelopsin-mediated sub-G1 phase arrest was caused by the overall downregulation of major cyclins, including cyclin D1, cyclin E1, cyclin A2, and cyclin B1, and upregulation of p21. In contrast, the S phase arrest by ampelopsin in K562 CML cells resulted from the downregulation of cyclin E1 and upregulation of p21. Meanwhile, ampelopsin did not result in a significant reduction in the expression of CDK proteins, including CDK4, CDK2, and CDK1, in both leukemia cell lines.

Ampelopsin also induced mitochondria-mediated apoptosis in HL60 and K562 leukemia cells. The mitochondrial pathway of apoptosis begins with the alteration of mitochondrial membrane permeability, thereby inducing the release of cytochrome c from the mitochondria to the cytosol [13]. Through interaction with apoptotic protease activating factor 1 (Apaf-1), cytochrome c can initiate the activation cascade of caspases. The complex promotes the activation of caspase-9, which in turn activates caspase-3, a key executioner of apoptosis. Consequently, caspases cause cell death by cleaving several cellular proteins, including poly (ADP-ribose) polymerase (PARP). In particular, this intrinsic pathway is regulated by Bcl-2 family members, which control mitochondrial membrane permeabilization [13,14]. The Bcl-2 family can be subdivided into anti-apoptotic members, such as Bcl-2 and Bcl-xL, and pro-apoptotic members, such as Bax and Bad. The accumulation of intracellular ROS also plays an upstream role in mitochondria-mediated apoptosis [13]. Our results showed that ampelopsin treatment induced the hallmarks of apoptosis through the intrinsic pathway in HL60 and K562 cells. This resulted in nuclear condensation, loss of mitochondrial membrane potential, increase in ROS generation, activation of caspase-9, caspase-3, and PARP, upregulation of Bax, and downregulation of Bcl-xL in both cell lines.

The MAPK/ERK, PI3K/AKT, and NF-κB signaling pathways participate in multiple cellular events, such as cell survival, proliferation, motility, differentiation, and apoptosis [15,16,37]. However, these pathways are frequently deregulated in many cancers, including leukemia, by mutations in upstream receptors, chromosomal translocations, or overexpression of signaling effectors [15,16,37]. Therefore, interrupting these signaling pathways may be an attractive approach to suppress proliferation and promote apoptosis of leukemia cells. In this study, ampelopsin effectively downregulated the AKT and NF-κB signaling, but not ERK1/2, in HL60 AML and K562 CML cells, suggesting that the inhibitory effect of ampelopsin on leukemia cell growth may be involved in the blockade of AKT and NF-κB signaling pathways.

In the current study, we further identified the effect of ampelopsin on the stemness of leukemia cells. Increasing evidence has demonstrated that leukemia stem cells (LSCs), which have self-renewal and differentiation abilities, can drive leukemia growth and relapse [38]. Several transcription factors, such as Oct4 and Sox2, and cell surface markers, such as CD44 and CD133, contribute to the maintenance and propagation of LSCs [38]. Our results showed that ampelopsin suppresses the expression of leukemia stemness markers, including Oct4, Sox2, CD44, and CD133, in HL60 and K562 cells. Accordingly, ampelopsin may be an attractive chemotherapeutic agent against leukemia since it regulates both apoptosis and stemness of leukemia cells.

Interestingly, ampelopsin exhibited higher susceptibility to HL60 AML cells than K562 CML cells, unlike imatinib, which is more sensitive in K562 cells than in HL60 cells. The HL60 cell line is derived from acute promyelocytic leukemia (APL), the M3 subtype of AML [45]. APL is caused by a chromosomal translocation that involves the fusion of the *PML* and *RARA* genes [5]. The PML/RARα protein produced by this fusion functions abnormally, causing the accumulation of excess promyelocytes in the bone marrow, thereby disrupting the formation of normal white blood cells and leading to APL. Several drugs targeting the PML/RARα oncoprotein, such as all-*trans* retinoic acid and arsenic trioxide, have been approved by the Food and Drug Administration (FDA) for the treatment of APL [9,10]. However, drug resistance is frequently observed [9,10]. Based on our results, as a single agent or in combination with the approved drugs, ampelopsin might contribute to overcoming the primary drug resistance that results in the progression and relapse of APL.

## 4. Materials and Methods

### 4.1. Materials

Ampelopsin (dihydromyricetin) and imatinib mesylate were purchased from Sigma-Aldrich (St. Louis, MO, USA) and dissolved in dimethyl sulfoxide (DMSO) at a concentration of 100 mM. RPMI-1640 medium and fetal bovine serum (FBS) were obtained from Corning Cellgro (Manassas, VA, USA) and R&D systems (Minneapolis, MN, USA), respectively. A dead cell apoptosis kit with Annexin V-FITC and propidium iodide (PI; cat. no. V13242) and tetramethylrhodamine ethyl ester (TMRE; cat. no. T669) were purchased from Invitrogen (Carlsbad, CA, USA). L-glutamine and antibiotics were purchased from Gibco (Grand Island, NY, USA) and Lonza (Walkersville, MD, USA), respectively. The CellTiter-Glo^®^ Luminescent Cell Viability Assay Kit (cat. no. G7570) was purchased from Promega (Madison, WI, USA). The Muse^®^ Cell Cycle Kit (cat. no. MCH100106) was purchased from Merck Millipore (Darmstadt, Germany). 4′,6-diamidino-2-phenylindole (DAPI) and 2′,7′-dichlorofluorescein diacetate (DCFH-DA) were purchased from Sigma-Aldrich (St. Louis, MO, USA). Antibodies against cyclin A2 (55 kDa; cat. no. 4656), cyclin E1 (48 kDa; cat. no. 4129), cyclin D1 (36 kDa; cat. no. 2922), cyclin B1 (55 kDa; cat. no. 12231), CDC2 (34 kDa; cat. no. 9116), CDK4 (30 kDa; cat. no. 12790), CDK2 (33 kDa; cat. no. 2546), p21 (21 kDa; cat. no. 2947), cleaved caspase-9 (37 kDa; cat. no. 9501), cleaved caspase-3 (17 kDa; cat. no. 9661), cleaved PARP (89 kDa; cat. no. 9542), Bax (20 kDa; cat. no. 2772), Bcl-2 (28 kDa; cat. no. 2872), Bcl-xL (30 kDa; cat. no. 2764), phospho-ERK1/2 (Thr202/Tyr204, 42,44 kDa; cat. no. 9101), ERK1/2 (42,44 kDa; cat. no. 9102), phospho-AKT (Ser473, 60 kDa; cat. no. 4060), AKT (60 kDa; cat. no. 9272), phospho-NF-κB (Ser536, 65 kDa; cat. no. 3033), NF-κB (65 kDa; cat. no. 8242), Oct4 (45 kDa; cat. no. 2750), CD44 (80 kDa; cat. no. 37259), CD133 (133 kDa; cat. no. 64326), Sox2 (35 kDa; cat. no. 3579), rabbit IgG (cat. no. 7074), and mouse IgG (cat. no. 7076) were purchased from Cell Signaling Technology (Danvers, MA, USA). Anti-β-actin (42 kDa; cat. no. ab6276) was purchased from Abcam (Cambridge, UK).

### 4.2. Cell Culture

Human HL60 AML and K562 CML cell lines were obtained from the Korean Cell Line Bank (Seoul, Korea). The human 267B1 prostate epithelial and MRC-5 fetal lung fibroblast cell lines were kindly provided by the Anticancer Agent Research Center at the Korea Research Institute of Bioscience and Biotechnology (Cheongju, Korea). The cells were cultured in RPMI-1640 medium supplemented with 10% FBS and 1% antibiotics and maintained at 37 °C in a humidified 5% CO_2_ incubator (Thermo Scientific, Vantaa, Finland).

### 4.3. Cell Proliferation Assay

Cell proliferation was determined using the CellTiter-Glo^®^ luminescent assay system. Briefly, cells (1 × 10^4^ cells/well) were seeded in a 96-white well culture plate and treated with ampelopsin (0−400 µM) for 24 or 48 h. Following the addition of 50 µL of the reaction mixture to each well, the culture plate was shaken for 2 min and incubated at room temperature in the dark for 10 min. The luminescent signal was measured using a multimode microplate reader (BioTek, Inc., Winooski, VT, USA). The IC_50_ values from the obtained data were analyzed using the curve-fitting program GraphPad Prism 5 (GraphPad Software, La Jolla, CA, USA).

### 4.4. Cell Cycle Analysis

Cell cycle analysis was performed using a Muse^®^ cell cycle kit, according to the manufacturer’s instructions. Briefly, cells (1 × 10^5^ cells/well) were plated in a 6-well culture plate and treated with ampelopsin (50 and 100 µM) and imatinib (5 µM) for 24 h. The cells were harvested and fixed with 70% ethanol at 4 °C for 6 h. After washing with PBS, the cells were stained with 200 µL of Muse cell cycle reagent and incubated in the dark at room temperature for 30 min. Cell cycle plots were analyzed using Muse cell analyzer and Muse analysis software (MuseSoft_V1.8.0.3; Luminex Corporation, Austin, TX, USA).

### 4.5. Western Blot Analysis

Cells were lysed using RIPA buffer (ATTO, Tokyo, Japan) supplemented with a protease inhibitor cocktail (Roche Diagnostics, Indianapolis, IN, USA) on ice. Protein concentrations were determined using the Pierce^®^ BCA Protein Assay Kit (Thermo Fisher Scientific, Inc., Rockford, IL, USA). Equal amounts of cell lysates were separated by 7.5–15% sodium dodecyl sulfate-polyacrylamide gel electrophoresis (SDS-PAGE) and then transferred to polyvinylidene difluoride (PVDF) membranes (EMD Millipore, Hayward, CA, USA) using standard electroblotting procedures. Blots were blocked in Tris-buffered saline with Tween-20 (TBST) containing 5% skim milk at room temperature for 1 h and immunolabeled with primary antibodies against cyclin A2, cyclin E1, cyclin D1, cyclin B1, CDC2, CDK4, CDK2, p21, cleaved caspase-9, cleaved caspase-3, PARP, Bax, Bcl-2, Bcl-xL, phospho-ERK1/2, ERK1/2, phospho-AKT, AKT, phospho-NF-κB, NF-κB, Oct4, CD44, CD133, Sox2 (dilution 1:2000), and β-actin (dilution 1:10,000) overnight at 4 °C. After washing with TBST three times, the membranes were incubated with horseradish peroxidase-conjugated anti-rabbit or anti-mouse (dilution 1:3000) secondary antibody for 1 h at room temperature. Immunolabeling was detected using an enhanced chemiluminescence (ECL) kit (Bio-Rad Laboratories, Hercules, CA, USA) according to the manufacturer’s instructions. The band density was analyzed using ImageJ software (version 1.5; NIH).

### 4.6. Apoptosis Analysis

Cells (1 × 10^5^ cells/well) were seeded in a 6-well culture plate and treated with ampelopsin (50 and 100 µM) and imatinib (5 µM) for 24 or 48 h. The cells were harvested and washed with phosphate-buffered saline (PBS). After resuspending with 100 µL of Annexin-binding buffer, the cells were stained with 5 µL of Annexin V-FITC and 1 µL of PI, according to the manufacturer’s instructions. Stained cells were analyzed by flow cytometry (CyFlow^®^ Cube 6, Sysmex, Kobe, Japan).

### 4.7. DAPI Staining

Cells (3 × 10^5^ cells/well) were seeded in a 12-well culture plate and treated with ampelopsin (50 and 100 µM) and imatinib (5 µM) for 24 h. The cells were fixed with 4% paraformaldehyde for 30 min. Nuclei were stained with 10 μg/mL DAPI for 15 min and washed with PBS. The nuclear morphology of the cells was observed using a 400× fluorescence microscope (Optinity KI-2000F, Korea Lab Tech, Seong Nam, Korea).

### 4.8. Mitochondrial Membrane Potential (MMP) Measurement

Cells (3 × 10^5^ cells/well) were plated in a 12-well culture plate. After treatment with ampelopsin (50 and 100 µM) and imatinib (5 µM) for 6 h, the cells were washed with PBS and stained with TMRE (100 nM) for 20 min. Images were obtained using a 200× fluorescence microscope (Optinity KI-2000F), and the fluorescence density was analyzed using ImageJ software (version 1.5; NIH).

### 4.9. Intracellular Reactive Oxygen Species (ROS) Measurement

Cells (3 × 10^5^ cells/well) were plated in a 12-well culture plate and treated with ampelopsin (50 and 100 µM) and imatinib (5 µM) for 6 h. The cells were fixed with 4% paraformaldehyde for 30 min and stained with DCFH-DA (10 μM) for 20 min. Images were obtained using a 200× fluorescence microscope (Optinity KI-2000F), and the fluorescence density was analyzed using ImageJ software (version 1.5; NIH).

### 4.10. Statistical Analysis

Results are expressed as the mean ± standard deviation (SD) from at least three independent experiments. Differences among groups were analyzed using analysis of variance (ANOVA) with the SPSS statistics package (SPSS 9.0; SPSS Inc.). Post-hoc analysis was performed using Tukey’s test. Statistical significance was set at *p* < 0.05.

## 5. Conclusions

In this study, we investigated the anticancer effects and underlying molecular mechanisms of ampelopsin, a flavanonol, on leukemia. Ampelopsin induced cell cycle arrest at the sub-G1 phase in HL60 AML cells but the S phase in K562 CML cells by regulating the expression of cyclins, CDKs, and CDK inhibitors differently in each leukemia cell. Ampelopsin also induced apoptosis in both leukemia cell lines through the mitochondria-mediated apoptotic pathway, including nuclear condensation, loss of mitochondrial membrane potential, increase in ROS generation, activation of caspase-9, caspase-3, and PARP, and regulation of Bcl-2 family members. Moreover, the antileukemic effect of ampelopsin was involved in the blockade of AKT and NF-κB signaling. Furthermore, ampelopsin suppressed the expression of leukemia stemness markers, including Oct4, Sox2, CD44, and CD133, in both cell lines, suggesting that ampelopsin exerts its chemotherapeutic effects by regulating both apoptosis and stemness of leukemia cells. Notably, ampelopsin exhibited higher susceptibility to HL60 cells than K562 cells. Therefore, ampelopsin may effectively suppress the progression and relapse of APL, the M3 subtype of AML.

## Data Availability

The data that support the findings of this study are available from the corresponding author upon reasonable request.
